# Fiber Embroidery of Self-Sensing Soft Actuators

**DOI:** 10.3390/biomimetics3030024

**Published:** 2018-09-04

**Authors:** Steven Ceron, Itai Cohen, Robert F. Shepherd, James H. Pikul, Cindy Harnett

**Affiliations:** 1 Department of Mechanical and Aerospace Engineering, Cornell University, Ithaca, NY 14850, USA; sc2775@cornell.edu (S.C.); rfs247@cornell.edu (R.F.S.); 2 Department of Physics, Cornell University, Ithaca, NY 14850, USA; ic64@cornell.edu; 3 Department of Mechanical Engineering and Applied Mechanics, University of Pennsylvania, Philadelphia, PA 19104, USA; pikul@seas.upenn.edu; 4 Department of Electrical and Computer Engineering, University of Louisville, Louisville, KY 40292, USA

**Keywords:** soft actuators, twisting, shape-changing, elastomer

## Abstract

Natural organisms use a combination of contracting muscles and inextensible fibers to transform into controllable shapes, camouflage into their surrounding environment, and catch prey. Replicating these capabilities with engineered materials is challenging because of the difficulty in manufacturing and controlling soft material actuators with embedded fibers. In addition, while linear and bending motions are common in soft actuators, rotary motions require three-dimensional fiber wrapping or multiple bending or linear elements working in coordination that are challenging to design and fabricate. In this work, an automatic embroidery machine patterned Kevlar™ fibers and stretchable optical fibers into inflatable silicone membranes to control their inflated shape and enable sensing. This embroidery-based fabrication technique is simple, low cost, and allows for precise and custom patterning of fibers in elastomers. Using this technique, we developed inflatable elastomeric actuators embedded with a planar spiral pattern of high-strength Kevlar™ fibers that inflate into radially symmetric shapes and achieve nearly 180° angular rotation and 10 cm linear displacement.

## 1. Introduction

Protrusible muscular organs, like a frog’s tongue or squid’s tentacle, use a combination of constricting muscles and inextensible fibers to rapidly elongate soft tissue appendages and catch prey [1]. A *Loligo pealei* squid’s tentacle can elongate by up to 80% in 40 milliseconds, while changing the tip trajectory to adjust for prey movement [2,3]. The bending, twisting, and elongation of a tentacle are controlled by tentacle extensor fibers, which are inextensible fibers arranged in circumferential and perpendicular arrangements relative to the tentacle’s long axis. 

Twisting and rotation are essential for endpoint control, working around obstacles, and carrying out rotating operations with objects like doorknobs and screwdrivers. Researchers have previously achieved rotation by inflating compliant materials that are more akin to soft tissue than to axles and wheels. The collapse of coupled soft structures produced local rotation [4], the unfolding of creases during inflation led to combined twisting and rotation of origami cylinders [5], and radial arrays of soft bending actuators were sequenced for continuous rotation [6]. Even closer to structures found in nature, lightweight and inherently safe robotic tentacles and limbs capable of rotation have been previously constructed from fabrics, with endpoint rotation achieved by bending inflated fabric tubes at joints. Approaches to bending joints include using a polymer mesh to control local stiffness [7,8], changing the fabric tension with cables on either side of the joint [9], and using gussets to vary the fabric extensibility on one side of the tube for a continuous tentacle-like limb [10]. Another broad category of fiber-based inflatable actuators is the McKibben actuator (reviewed in [11]), which uses fibers wrapped around cylindrical elastomeric tubes to control the degree of contraction or elongation of the actuator during the inflation of the elastomeric chamber. McKibben actuators have long been used as artificial muscles in robotic arms. McKibben tubes have also been embedded within a membrane for contractile motion and twisting motion of up to 30° to assist the pumping of animal hearts [12]. Not only can these devices work as linear actuators, but the spacing between fibers, their relative angle, and the elastomer stiffness can be adjusted to cause bending or twisting [13,14]. The fiber angle and elastomer stiffness determine the degree of actuator twist [14]. However, the fabrication process for realizing twisting is difficult because the fibers are helically wound by hand around an elongated, elastomeric chamber.

The key difference between the above inflatable actuators and the rotating membrane presented in this work is that our fiber patterns are fabricated in a plane rather than on a cylinder or other three-dimensional (3D) structure. Planar fabrication makes high speed assembly, alignment, and cutting operations possible without the need to grasp and orient a complex shape; it is the foundation of both the semiconductor and garment assembly industry. Textile equipment for sewing, weaving, knitting, and embroidery produces objects from fibers at high-speed and at low enough cost to satisfy a price-sensitive market. With fabric clamped in a planar format, industrial embroidery machines typically insert 20 stitches per second, while consumer-grade machines do 5–10 stitches per second. 

Merging soft, inflatable fiber-reinforced actuator functionality [15] with textile fabrication methods enables rapid soft actuator fabrication with a diverse range of functional fibers and fabrics. In one such example, yarn coated with an electroactive polymer was custom-knitted into textiles and inserted in an electrolyte solution, producing localized fabric contractions [16]. In another, our team showed that patterned cutouts from inextensible fiber sheets embedded in silicone membranes could control the 3D shape of inflated silicone structures [17]. 

**Figure 1:**
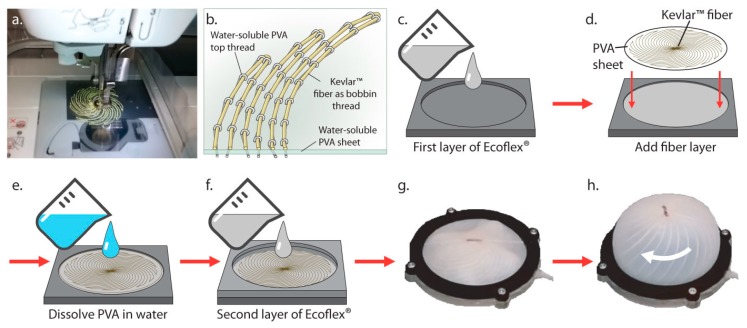
A pattern of inextensible fibers made in a water-soluble plastic sheet using (**a**,**b**) an embroidery machine (**c**–**f**) is embedded in a silicone membrane (**g**,**h**) causing vertical and rotational displacement. PVA: Poly(vinyl alcohol).

Machine embroidery solves the problem of orienting fibers in complex, reproducible two-dimensional (2D) patterns for transfer into other materials. In this work, we have developed actuators that reversibly twist and transform from a 2D plane into axisymmetric 3D shapes upon inflation. The silicone disc actuators are embedded with Kevlar™ fibers spirally patterned by a computer-automated embroidery machine. Silicone disc actuators with spiral patterns have previously achieved dome-like bending using spiral air chambers [18] and self-sensing using a spiral-patterned liquid metal resistive/capacitive sensor [5]. They have also been proposed as a way to change the handedness of spiral microantennas [19]. While those works did not investigate in-plane twisting of the discs during inflation, they were similar to our work in that the spiral-patterned materials (air, liquid metal, and a thin metal film, respectively) had a different modulus than the elastomer. The Kevlar™ polyaramide fibers in this paper have a high tensile strength (3620 MPa) thanks to hydrogen bonds between adjacent polymer chains. The fiber patterns controlled the final inflated shapes and maximum rotations, up to 170° for the inflated membranes shown in Figure 1. The fabrication process was simple, fast (2–3 min to form the fiber pattern), and used water-soluble thread and stabilizer to lock complex fiber shapes into solution-cured elastomers. Additionally, by placing stretchable optical waveguides over the inflating elastomeric discs, we were able to sense the optical signal output corresponding to the degree of rotational actuation of specific fiber-patterned membranes. These actuators present a new approach for the scalable fabrication of synthetic skins that combine local actuation and sensing.

## 2. Materials and Methods

This study focused on spiral fiber patterns that could be parametrized by a few variables. Threads radiating in a spiral from the center of an inflatable membrane can be expected to induce twisting, because when the threads are inextensible compared with the elastomer, they must straighten out, untwisting the spiral, in order to reach the center of the membrane as it rises. 

### 2.1. Spiral Pattern Design for Embroidered Actuators

Spiral patterns were created with parametric Equations (1) and (2) that consider *k*, the number of times each spiral wraps around the center, and *n*, the total number of spirals emanating from the center. Both *r* (Equation (1)) and *θ* (Equation (2)) increase linearly as the parameter *t* ranges from 0 at the center to 1 at the edge, forming a family of interleaved Archimedes spirals,

(1) $$r\left(t\right)=r_{max}t$$

(2) $$\theta \left(t\right)=2\pi kt+\frac{2\pi \left(m-1\right)}{n},$$

where *r_max_* is the outer radius of the pattern, and *2π*(*m* − 1)/*n*, where *m* ranges from 1 to *n*, is the angular offset of the *m*th spiral to achieve equal angular spacing.

The embroidery machine sewed each pattern with one continuous thread, using the *x*,*y* coordinates generated by the parametric Equations (3) and (4). We obtained the best performance by joining pairs of spirals across the center. There were even numbers of spirals in each pattern, and we wrote the coordinates in a sequence that connected each spiral with the one on the opposite side,

(3) $$x\left(t\right)=r_{max}\;t\;cos\left(2\pi kt+\frac{2\pi \left(m-1\right)}{n}\right)$$

(4) $$y\left(t\right)=r_{max}\;t\;sin\left(2\pi kt+\frac{2\pi \left(m-1\right)}{n}\right).$$

The coordinates for the embroidery design were generated in MATLAB (MathWorks, Inc., Natick, MA, USA) to conform to the EXP embroidery file format. Embird conversion software (Balarad, S. R. O., Prešov, Slovakia) translated the EXP file format to the PES file format required by the embroidery machine.

### 2.2. Membrane Fabrication

The procedure for fabricating a fiber-embedded elastomer is shown in Figure 1. A water-soluble poly(vinyl alcohol) (PVA) sheet (Ultrasolvy stabilizer, Sulky of America, Inc., Punta Gorda, FL, USA) was embroidered with a spiral fiber pattern (Brother PE-525 embroidery machine, Brother Industries, Ltd., Nagoya, Aichi, Japan) using water-soluble PVA thread (Wash-A-Way, YLI Corp., Rock Hill, SC, USA) in the needle, and 23 lb Kevlar™ fiber (0.0081 inch diameter, The Thread Exchange, Weaverville, NC, USA) as bobbin thread (Figure 1a,b). The process also worked using 100% polyester sewing thread as the needle thread, instead of Wash-A-Way. A two-part liquid-cure silicone (Ecoflex^®^ 00-10, Smooth-On, Inc., Macungie, PA, USA) was chosen for the stretchable membrane material, because it was soft enough to achieve maximum rotation at low (<3 psi) pressures that did not cause leaking around the seal. Equal amounts of Ecoflex^®^ 00-10 (A) and (B) were thoroughly mixed, and 20 g of the mixture was poured into a plastic mold with a 90 mm diameter and 2 mm depth (Figure 1c). The silicone was degassed in a vacuum chamber. The fiber pattern was embedded Kevlar™ side down into the still-uncured Ecoflex^®^ 00-10 (Figure 1d). A similar method has been used by others to insert fine wires into elastomer castings using a sewing machine without a programmable path [20]. In our work, the machine guided the thread to each *x*,*y* coordinate as shown in Figure 1a. The silicone was then cured in a 70 °C oven for 30 min. After curing, the water-soluble plastic was washed away in smooth-running tap water (Figure 1e) with care to avoid displacing the embedded fibers. 

In the final step, the embedded Kevlar™ fibers were covered with a top layer of silicone by pouring a second 20 g layer of Ecoflex^®^ 00-10 over the mold (Figure 1f), degassing it for five minutes, and then curing it again in the oven. At the end of the process, the fiber pattern was embedded at the mid- thickness of a 3 mm thick, ≈90 mm diameter Ecoflex^®^ circle. Optionally, after curing the top Ecoflex^®^ layer, a stretchable optical fiber [21] was placed across the spiral pattern through its center. Ecoflex^®^ was dispensed on top of the fiber in a thin layer and cured to attach it to the surface. Thread-embedded silicone membranes were removed from the mold and clamped for testing. Figure 1 shows one of the actuators before (Figure 1g) and after (Figure 1h) inflation. Inflation produces both rotational and vertical displacement of the membrane.

### 2.3. Testing Methods

We used an image analysis program (Adobe Photoshop, Adobe Systems, Inc., San Jose, CA, USA) to measure the rotational motion and 3D deformations induced by the thread pattern and inflation pressure; specifically to measure angles and *x*,*y* coordinates in images. The actuators were characterized by maximum degrees of rotation, torque output, and shape profile. A membrane with an embedded optical fiber was also investigated as a rotation sensor toward a control system for actuation.

#### 2.3.1. Measuring Inflation of Soft Actuators

A pressure-regulated pump (EFD Inc., East Providence, RI, USA) was used to inflate the soft membranes throughout the experiments. Prior to inflation, an actuator was placed on top of a piece of laser-cut acrylic with an inner chamber of approximately 2 mm in depth. A second piece of acrylic with a ≈90 mm diameter hole in the middle was placed on top of the membrane. The two pieces of acrylic were screwed together around the membrane edge to create enough clamping force to inflate the membrane. The pressure differential (Δ*P*) between the inner bladder and atmosphere could be set in the range Δ*P* = 0 to Δ*P* = 3 psi without leaking.

#### 2.3.2. Rotational Motion Measurements

Prior to inflating a soft actuator, a line ≈3 cm long was drawn at the center of the membrane with a marker. During inflation, a digital camera (EOS Rebel T3i, Canon Inc., Tokyo, Ota, Japan) recorded top-view videos over the center of the membrane to capture the actuator’s twisting motion, and then the first and final frames of each recording were isolated and analyzed to measure the total twisting angle. 

#### 2.3.3. Shape Characterization

**Figure 2:**
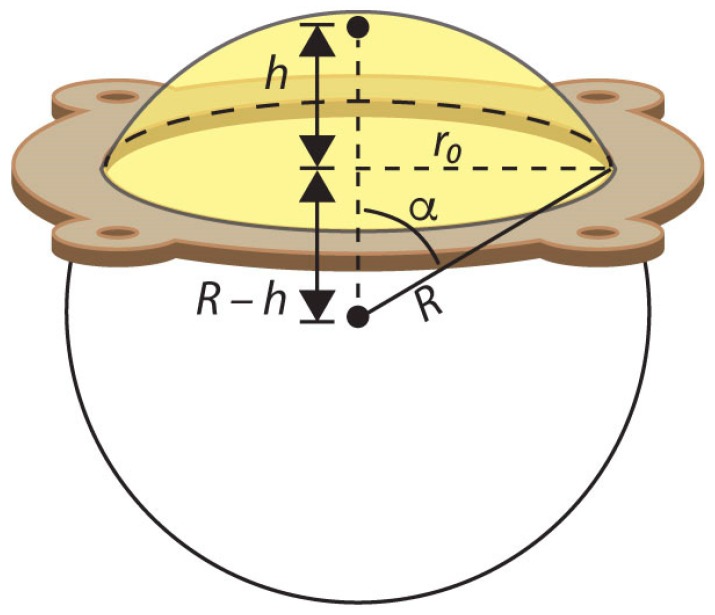
Spherical cap coordinate system used in energy balance model for an inflating silicone membrane (yellow region). *h*: Center height; *R*: Radius of the spherical cap; *r*_0_: Base radius of the membrane; α: Zenith angle.

We also recorded the membranes’ inflations using side-view video, and 10 evenly spaced frames from the recording were isolated for image analysis. For each frame, we selected ten approximately evenly spaced points along an inflated membrane’s left half surface and used their locations with respect to the highest point at the center of the membrane. These ten points were used to characterize how closely the shape of each membrane matched the spherical cap model illustrated in Figure 2; we determined the radius *R* of a spherical cap matching the membrane’s base radius, *r*_0_, and its measured center height *h*. A polygon-based shape metric (see Supplementary Methods, Equation (S1)) was used to compare the measured profile shape to the corresponding spherical cap. 

#### 2.3.4. Torque Measurements 

Torque was measured using a 14 cm long, flat, lightweight (≈0.4 g) wooden stick glued with Loctite^®^ 404 to the center of the actuator (Supplementary Figure S1). The end of the stick was pressed down on a scale to measure the torque produced at increasing angles at pressures up to 3 psi.

#### 2.3.5. Fiber Optic Lamination and Testing

A stretchable fiber optic waveguide (for details, see Supplementary Information in [21]) was placed across an embedded fiber pattern, and then cast under a poured layer of Ecoflex^®^ 00-10. Fiber ends were connected to an infrared light-emitting diode (LED) and an amplified photodiode (TSL-12, ams-Taos Inc., Plano, TX, USA).

## 3. Actuator Model

We used the energy balance between the pressurized gas and membrane strain to predict the inflated membrane shapes. The inextensible fibers force the membrane to only stretch in the direction perpendicular to the fiber alignment. The fibers can bend freely and, when they unwind, the compliance allows the center of the membrane to rise up. At a given pressure, the energy stored in the stretched and sheared silicone balances the energy lost by the gas as the volume increases. The membrane minimizes the amount of stored strain energy in the system by finding a shape with the smallest amount of surface area (stretch) and rotation (shear) that will enclose the volume, subject to the constraint that the Kevlar™ threads cannot stretch. Our semi-empirical model approximates the shape of the membrane as a spherical cap, finds the radius that achieves the energy balance at each pressure, and links the twist angle to the radius using the geometry of the spiral projected onto the spherical cap.

### 3.1. Energy Balance

The energy stored in the system can be modeled as

(5) $$U=\gamma A\left(\frac{A-A_{0}}{A_{0}}\right)+\frac{1}{2}\kappa \theta^{2}-PV.$$

where γ is a strain energy coefficient having units of J m^−2^, *A* is the surface area of the inflated thin membrane, *A*_0_ is the surface area of the flat membrane, *κ* is a torsional spring constant for the membrane, *θ* is the rotation angle, *P* is the inflation pressure, and *V* is the enclosed volume.

Because the membrane takes on a rotation angle *θ* that minimizes the total energy at a given pressure *P*,

(6) $$\frac{dU}{d\theta }=\gamma \left(\frac{2A}{A_{0}}-1\right)\frac{dA}{d\theta }+\kappa \theta -P\frac{dV}{d\theta }.$$

Rather than extracting *θ*(*P*), the twist angle as a function of pressure, from Equation (6), it is more straightforward to solve for *P* as a function of rotation angle *θ*, as follows:

(7) $$P\left(\theta \right)=\frac{\gamma \left(\frac{2A}{A_{0}}-1\right)\frac{dA}{d\theta }+\kappa \theta }{\frac{dV}{d\theta }}.$$

We can invert the measured rotation vs. pressure data and use it for fitting Equation (7). However, the area *A* and volume *V* still need to be defined in terms of *θ*.

### 3.2. Spherical Cap Approximation

Because an inflating membrane without embedded fibers takes on a spherical cap shape [22], we use the spherical cap as an approximate shape to connect the membrane area *A* and volume *V* to the rotation angle *θ*, so Equation (7) can be solved for pressure. Later (Section 4.2), we will validate this assumption using image analysis. The surface area of a spherical cap (Figure 2) is

(8) $$A=\pi \left({r_{0}}^{2}+h^{2}\right),$$

and its volume is

(9) $$V=\frac{\pi h}{6}\left(3r_{0}{}^{2}+h^{2}\right).$$

In the membrane system, *r*_0_ is the fixed radius of the testing plate (0.038 m) and *h* is the height of the membrane center above the plate, which varies with pressure.

### 3.3. Spiral Mapping onto Spherical Cap

The spiral is projected onto the spherical cap using a method that preserves the relative radial distances between features [23]. We map the radial coordinate *r* of the flat spiral (Equation (1)) to an arc length along a meridian passing through the center of the sphere, *r* > *R*α, where α is the zenith angle on the spherical cap (α = 0 at the center) and *R* is the radius of the sphere (see Supplementary Figure S2 for an example). The spiral must also untwist by *θ* to make its own arc length match its initial value, because the threads cannot stretch, and that gives the connection between *h* and *θ*. A zero-finding method is used to solve for the twist angle *θ* that makes the projected spiral arc length match up with the original flat arc length at a given center height *h.*

The twist angle *θ* for a given center height *h* depends on the spiral pattern wrapping number *k*. With an array of *h* values calculated for the different values of *θ*, it is possible to numerically calculate *dh*/*dθ*. Equation (7) for the pressure was rewritten using derivatives with respect to *h*, and the terms *A*, *dA*/*dh*, and *dV*/*dh* were computed using the spherical cap geometry in Equations (8) and (9) to obtain an expression for the pressure as a function of rotation angle, up to a few coefficients,

(10) $$P\left(\theta \right)=\frac{\gamma \left(\frac{2A}{A_{0}}-1\right)\frac{dA}{dh}\frac{dh}{d\theta }+\kappa \theta }{\frac{dV}{dh}\frac{dh}{d\theta }}.$$

### 3.4. Values for Coefficients

#### 3.4.1. Strain Energy

The area term in the energy expression (Equation (5)) has a proportionality constant γ that connects the change in membrane surface area to an energy cost. In the Yeoh model [24], the energy density u in a strained material is

(11) $$u=\sum_{i=1}^{n}C_{i}{\left(I_{1}-3\right)}^{i},$$

where $C_{i}$ are the Yeoh coefficients, $I_{1}=\lambda_{1}{}^{2}+\lambda_{2}{}^{2}+\lambda_{3}{}^{2}$, and λ=ε+1 is the principal stretch along each direction. Looking only at the first term in Equation (11), *u* = $C_{1}\left(I_{1}-3\right),$ one can write out the $\lambda^{2}$ terms as (*L_x_*/*L_x_*_0_)^2^ and (*L_y_*/*L_y_*_0_)^2^, or for equibiaxial stretching, *A*/*A*_0_. Setting $\lambda_{3}$ to 1 means the membrane thickness does not change during inflation, a simplification made to get an estimate for the value of γ in the first-order energy balance model. Re-assembling the first term in Equation (11),

(12) $$u=C_{1}\left(\left(\frac{2A}{A_{0}}\right)+1-3\right)=\frac{2C_{1}\left(A-A_{0}\right)}{A_{0}}.$$

The first Yeoh coefficient *C*_1_ for Ecoflex^®^ 00-30 was measured by others [25] to be 1.27 × 10^−2^ MPa; it is likely smaller for the softer Ecoflex^®^ 00-10 silicone we used. The total strain energy in the membrane is estimated by multiplying the strain energy density in Equation (11) by the volume *tA* of the thin membrane material, where the thickness *t* is ≈3 mm and the surface area *A* is given by the spherical cap Equation (8),

(13) $$u_{strain}=\frac{2C_{1}tA\left(A-A_{0}\right)}{A_{0}}.$$

By comparing Equation (13) with the first term of the energy expression (Equation (5)), the coefficient γ should be in the neighborhood of ≈2*C*_1_*t* or 76 J m^−2^ if Ecoflex^®^ 00-30 were used. However, a smaller γ value of 30 J m^−2^ better matched the experimental data from the softer Ecoflex^®^ 00-10 membranes.

#### 3.4.2. Shear Energy

The torsional spring constant κ was found to be in the range of 0.06 N m rad^−1^ in the torque measurements; experimental details are provided in Section 4.3. 

## 4. Results and Discussion

The membranes were inflated and evaluated for their rotation angle, torque, shape, and fiber optic light transmission as a function of pressure, and results were compared with the spherical cap model.

### 4.1. Rotation Angle vs. Pressure

**Figure 3:**
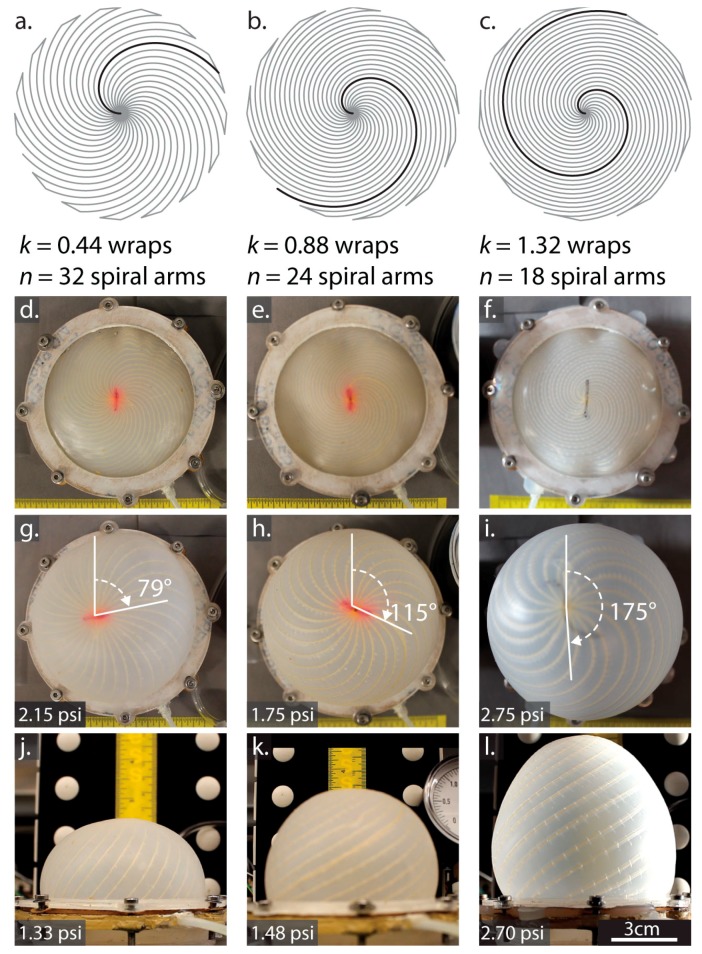
Layout and implementation for three different spiral designs (**a**–**c**) Embroidery layout for spiral patterns with three different wrap numbers k and similar thread densities. (**d**–**f**) Uninflated top views. (**g**–**i**) Inflated top views near the maximum rotation value for each actuator. (**j**–**l**) Side views used for comparing inflated shapes to the spherical cap model. These three designs were fabricated and inflated. As pressures increased, the membranes approached a maximum rotation angle (Table 1) determined by the wrap number *k*.

The columns in Figure 3 show the spiral layout, a side view, and top view photos at increasing pressures for membranes with wrap numbers (*k* from Equation (2)) of 0.44, 0.88, and 1.32, respectively.

We were able to double the final rotation angle by increasing the wrap number *k* from 0.44 turns to 1.32 turns. However, increasing *k* reduces spacing between fibers, limiting *n*, the number of spiral arms that would fit in the membrane. At *n* = 24 spirals and *k* = 1.32, for example, the embroidery machine was not able to complete the process without sewing over the adjacent Kevlar™ fiber, so the number of spirals was decreased to 18 for these actuators. Rotating and shape-changing occurred even with the lower number of spirals.

**Figure 4:**
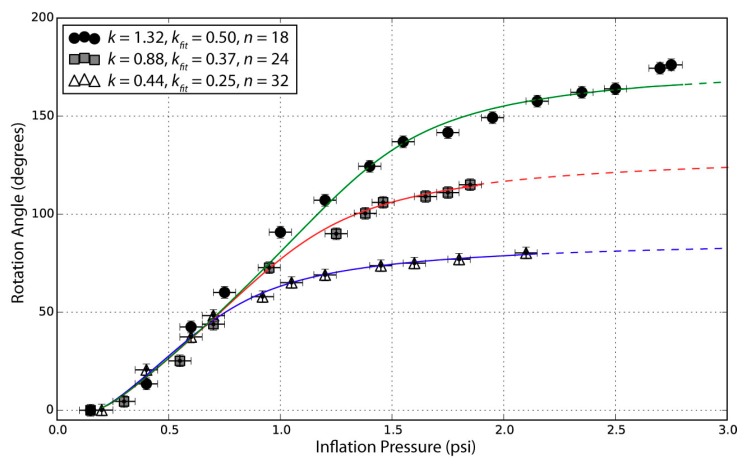
Rotation vs. pressure for an individual test of each of the three spiral designs in Figure 3, along with the spherical cap energy–balance model using torsional spring constant κ = 0.06 N m, strain energy coefficient γ = 30 J m^−2^, and base radius *r*_0_ = 0.038 m. Dashed lines indicate where the model extends beyond measured inflation pressures.

The theoretical absolute maximum rotation in degrees occurs when the spirals become straight lines radiating from the center. For the three designs in Table 1, the maximum possible rotations are 158°, 317°, and 475° in order of increasing *k*. Although the experimental results in Table 1 do not reach these absolute maximum angles, instead leveling off with pressure (Figure 4) as the silicone resists stretching and shearing. To increase the maximum rotation angle, the silicone might be changed to a softer material, or the spacing between the fibers might be increased. These adjustments, however, reach a limit because with softer silicone and greater spacing, energy transfers to volumetric expansion of the silicone between the threads. The spherical cap model includes strain energy stored in the silicone, but does not account for the effects of fiber spacing. The three *k*,*n* combinations shown in Figure 4 all have similar fiber spacing in the optimal region, between one and two times the membrane thickness, where fibers are dense enough to prevent the silicone from bulging between them, yet sparse enough to still allow twisting. Supplementary Figure S3 can give some insight on the relationship between *k*,*n*, and fiber density.

In Figure 4, for Δ*P* < 0.75 psi, the rotation for all three membranes was similar, with a slope of ≈90° per psi. Above 0.75 psi, the *k* = 0.44 membrane diverged and its rotation angle began to level off, followed by the *k* = 0.88 membrane near 1 psi. At Δ*P* ≥ 3 psi, the clamping force at the edges was insufficient, and the membranes began to slip out of the fixture. 

The spherical cap model, plotted as lines through data points in Figure 4, captures the general shape of the twist vs. pressure curves, as well as the increase in final twist angle with *k*-value. The least squares error-minimizing fits in Figure 4 were obtained using smaller *k*-values (0.25, 0.37, and 0.50) than the pattern (0.44, 0.88, and 1.32, respectively). This discrepancy in actual vs. best-fit *k*-values could be caused by the dense threads in the middle reducing the number of usable wraps as the threads occupy a large portion (50%) of the membrane surface within ≈1cm of the center. Also, energy stored in lateral compression of silicone in front of the threads was not captured in the model, but at pressures greater than 1 psi, we observed air pockets opening behind the threads as the silicone resisted twisting (Supplementary Figure S4); thicker threads might reduce this effect by providing a larger bonding surface. Making a reliable interface between soft, highly compressible materials like silicone and less-compressible materials like the Kevlar™ fibers in this paper is still one of the fundamental challenges in soft robotics fabrication [17]. Materials solutions, for example, using adhesives or a porous, loosely twisted fiber with greater bonding area, would likely increase the rotation angle, lifting the curves in Figure 4 to match higher *k*-values by pulling on the silicone instead of opening the air gap behind it. 

### 4.2. Actuator Shape Compared with Spherical Cap Model

**Figure 5:**
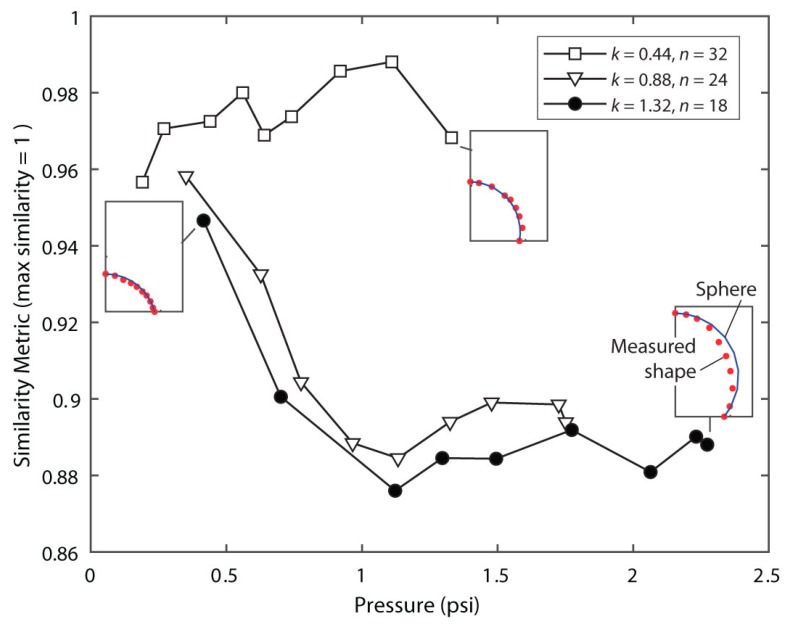
Sphere similarity metric vs. pressure, and measured vs. ideal spherical cap shape for three cases. A metric of 1 means the shape has 100% overlap with the spherical cap.

In this section, the validity of the spherical cap model is evaluated from actuator images. Cross-sections of the measured shape in comparison with the ideal spherical cap are shown in Supplementary Figure S5. In Figure 5, a polygon-based similarity metric (details in Supplementary Methods, Equation (S1)) quantifies the departure from the spherical cap shape with increasing pressure. In this metric, a score of 1 indicates 100% overlap of the measured inflated shape with a spherical cap. Before inflation begins, the pressure differential between the inner bladder and atmosphere is Δ*P* = 0 and the membranes are slack, making the surface non-spherical. After the onset of inflation, the observed shapes do resemble spherical caps (similarity coefficient >0.9) for all three designs. As shown in Figure 5, the spherical cap shape used in the model is an excellent match for the measured *k* = 0.44 membrane shape across its full actuation range, while the higher twist *k* = 0.88 and *k* = 1.32 designs take on a more conical appearance (Figure 3l) above Δ*P* = 1 psi. 

### 4.3. Torque Exerted by the Actuator

**Figure 6:**
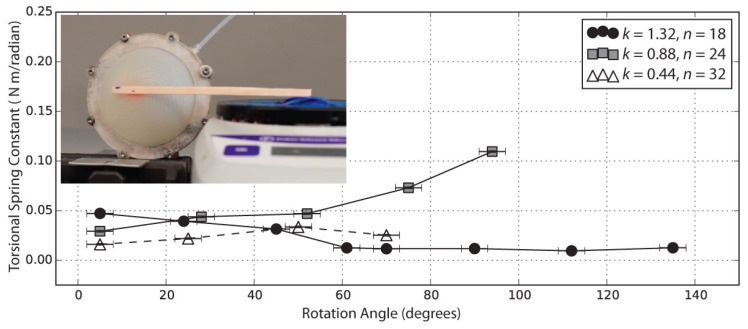
Torsional spring constant measured over the rotational range of three membranes with *k* = 0.44, *k* = 0.88, and *k* = 1.32.

The maximum torque measured by the method of Supplementary Figure S1 was 0.016 N m, attained by the *k* = 0.88 actuator. This torque would be sufficient to push a typical 40 gm tactile switch, like those found on microwave ovens and other household appliances, with a 4 cm lever. In Figure 6, the measured value for the torsional spring constant κ varies with angle, but it is in the 0.01–0.10 N m rad^−1^ neighborhood. For the model in Figure 4, a good match to the experimental data was obtained with a torsional spring constant κ of 0.06 N m rad^−1^, consistent with the experimental results. At the higher end of the rotation range for the highest-twist actuator, the contact area between the actuator and the wooden stick began to decrease as the actuator took on a more cone-shaped profile (Figure 3l); debonding may explain why the *k* = 1.32 actuator exerted a decreasing amount of torque at higher angles.

### 4.4. Optical Detection of Actuator State

**Figure 7:**
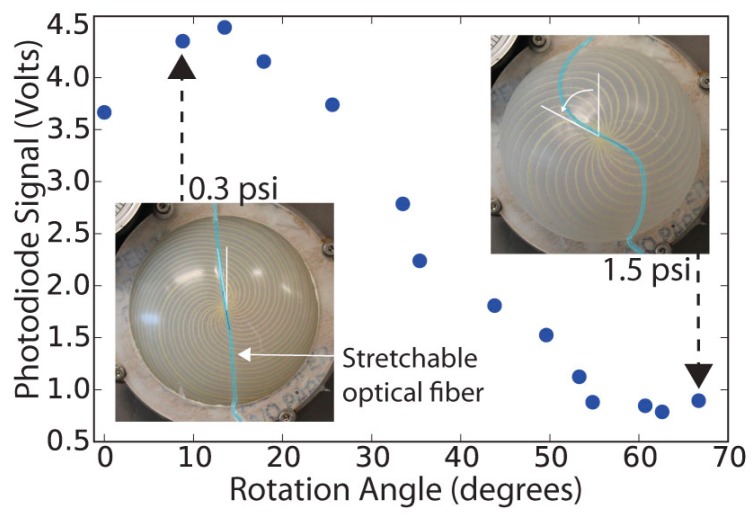
Optical signal as a function of rotation angle for a membrane with an embedded spiral pattern of *n* = 24 and *k* = 0.88.

Figure 7 shows the intensity signal output versus angle when a membrane with embedded stretchable optical fiber is inflated; this signal is plotted versus pressure in Figure S6.

At small angles, the fiber optic showed an increasing signal, likely because the membrane is still slack at very low pressures, causing bending losses from the fiber. As the pressure and rotation angle increase, the signal reaches a maximum. With further inflation, it begins to stretch, producing a monotonically decreasing signal between 15° and 50°. The inset in Figure 7 shows that the polyurethane-core, silicone-clad fiber is soft enough to follow the bending and stretching of the soft silicone surface, in contrast to the Kevlar™ threads that rotate in the opposite direction. Because the silicone is softer than the optical fiber, a small amount of distortion can be seen in the upper right inset of Figure 7, where the optical fiber crosses the Kevlar™ threads, showing the potential for a single material to add both sensing and mechanical actuation capability. Stretchable constraining materials were not included in the spherical cap model, which assumed the constraining fibers were completely inextensible. To accommodate stretchable fibers, an arc length-dependent spring energy term (^1^/_2_ *k_f_*Δ*s*^2^, where Δ*s* is the difference between the original and stretched arc length of the spiral arm, and *k_f_* is the spring constant of the fiber) could be added to the energy balance in Equation (5); the stretched arc length would be obtained from the spherical cap radius that balances the equation. A stretchable constraining material would likely yield smaller rotation and more volumetric expansion, as in [5], where 2D stretchable fabric inserts in silicone membranes led to both texture changes and volumetric expansion during inflation.

## 5. Conclusions

We have developed a method for programming the shape and rotation of surfaces by embroidering inextensible Kevlar™ fibers into soft silicone sheets. These actuators could rotate nearly 180° at 2.75 psi, adopt 3D axisymmetric shapes, and produce 0.016 N m torques. We developed a model that balanced the energy stored in the stretched and sheared silicone-fiber composite with the energy stored in compressed gas to predict the final actuator rotation angle. The embroidery-based fabrication process was simple, fast, and able to program complex patterns into silicone sheets using water soluble thread and stabilizer. In addition, we installed stretchable optical fiber sensors into the silicone sheets that detected the rotation angle, demonstrating the ability to embed fibers with different sensing and actuating functions. Improvements in the maximum composite material strain or in new fiber geometries would allow for higher rotation angles and increased torque. Additionally, embedding active fibers would enable controlled transformation between shapes.

In the biological world, oriented active fibers are responsible for motion over a wide range of scales, from separating genetic material during cell division, to organizing complex motions in human-scale muscular structures. The technological implications for this work are extensive and include the large area integration of textiles into soft synthetic skins for controlling the shape of intelligent catheters [26], improving aerodynamic efficiency in airplane wings [27], wearable sensors, soft robotic design [13,14,15,28], and controllable surface actuators that rotate and manipulate compliant external shapes [29].

## Figures and Tables

**Table 1:** Maximum rotation for three sets of design parameters.

*k*	*n*	Maximum Observed Rotation Angle (Degrees)	Pressure at Maximum Rotation (psi)
0.44	32	80	2.1
0.88	24	115	1.85
1.32	18	176	2.75

*k*: Wrap number; *n*: Number of arms.

## Supplementary Materials

The following are available online at http://www.mdpi.com/2313-7673/3/3/24/s1, Figure S1: Method for measuring the torsional spring constant κ of pressurized fiber-embedded membranes, Figure S2: An example of a *n* = 32, *k* = 0.44 spiral mapped onto a spherical cap, Figure S3: Percentage of membrane that has fiber spacing between one and two times the membrane thickness, for different values of wrap number *k* and number of spiral arms *n*, Figure S4: Example of air pockets opening behind threads at high pressures from *k* = 1.32, pressure = 1.5 psi, actuator at 132° rotation, Figure S5: Measured and ideal spherical cap cross-sections of inflated membranes for three *k*-values at increasing pressures (*p*) in psi, Figure S6: Fiber optic intensity signal vs. pressure for *k* = 0.88, *n* = 24 with straight stretchable optical fiber, Equation (S1): Shape similarity metric, Video S1: Side view of inflating actuator is available on Zenodo: http://doi.org/10.5281/zenodo.1408267, Video S2: Top view of inflating actuator is available on Zenodo: http://doi.org/10.5281/zenodo.1408263.
