# Supplementary material for: Fiber Embroidery of Self-Sensing Soft Actuators
Source: Biomimetics (Basel). 2018 Sep 4;3(3):24. doi: 10.3390/biomimetics3030024 (PMC6352685; doi:10.3390/biomimetics3030024)
Supplement: Supplementary file 1 [file biomimetics-03-00024-s001.pdf]

Biomimetics **2018**, *3*, 24

Supplementary Materials: Fiber Embroidery of Self-Sensing Soft Actuators

Steven Ceron [1], Itai Cohen [2], Robert F. Shepherd [1], James H. Pikul [3] and Cindy Harnett [4,*]

[1] Department of Mechanical and Aerospace Engineering, Cornell University, Ithaca, NY 14850, USA; sc2775@cornell.edu (S.C.); rfs247@cornell.edu (R.F.S.)
[2] Department of Physics, Cornell University, Ithaca, NY 14850, USA; ic64@cornell.edu
[3] Department of Mechanical Engineering and Applied Mechanics, University of Pennsylvania, Philadelphia, 19104 PA, USA; pikul@seas.upenn.edu
[4] Department of Electrical and Computer Engineering, University of Louisville, Louisville, KY 40292, USA
* Correspondence: cindy.harnett@louisville.edu; Tel.: +1-502-852-0689

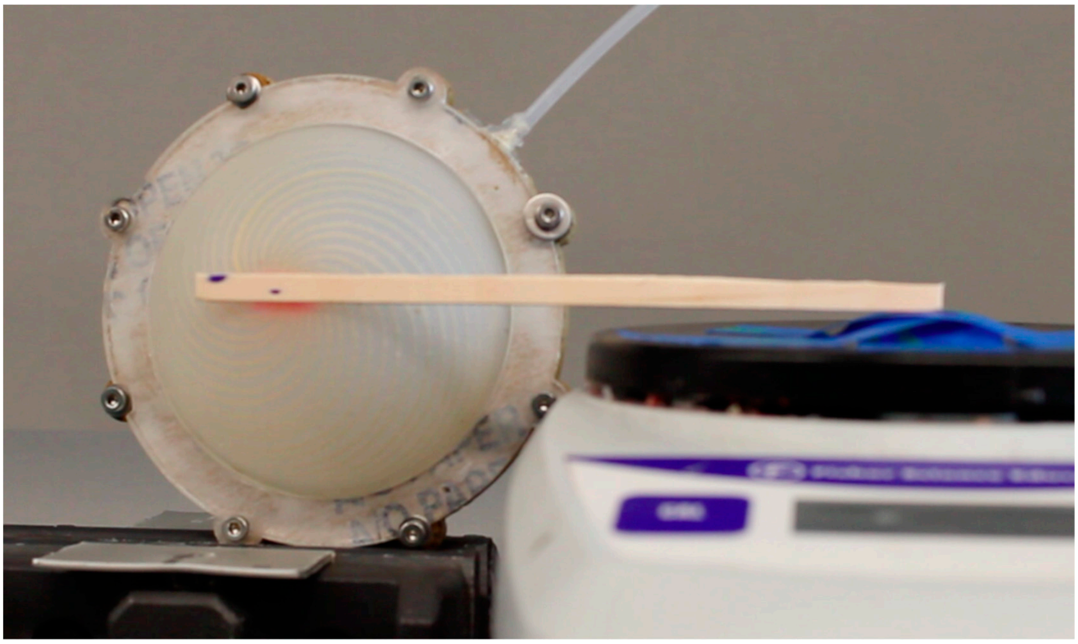

Figure S1. Method for measuring the torsional spring constant κ of pressurized fiber-embedded membranes. A glued-on stick (Loctite 404) exerted force on a scale. To measure the spring constant κ, the membrane was inflated to a fixed pressure, and was clamped so that the stick touched the scale (force = 0) at an angle 10 degrees below the horizontal. The scale was raised until the stick became horizontal, and the force F on the scale was measured. The torque $\tau = F \times$ stick length. Torsional spring constant $\kappa = \tau / \Delta\theta = F \times$ stick length/10°. We measured the value of κ at different pressures to check for variations across the rotation range.

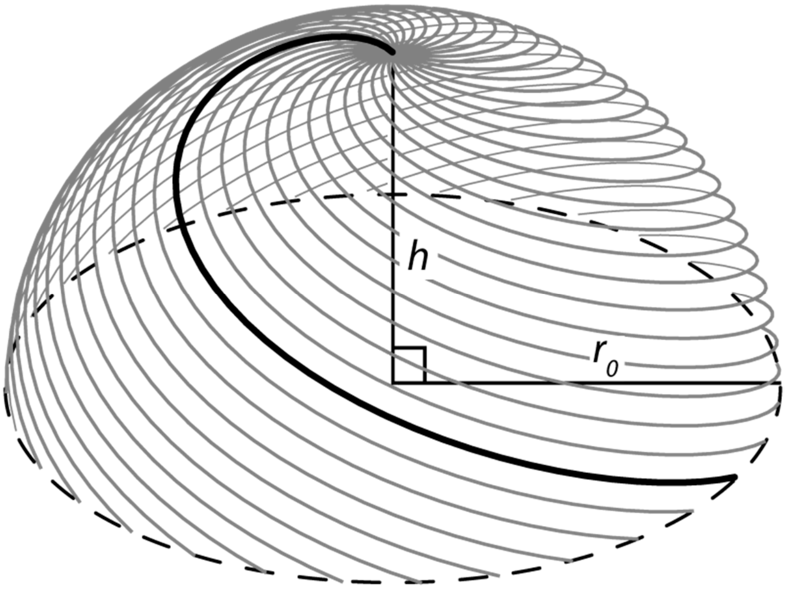

Figure S2. An example of a $n = 32$, $k = 0.44$ spiral mapped onto a spherical cap. During inflation, the center height of the cap increases from 0 to h, and the arc length of each spiral arm is preserved by rotating the cap by θ. This example is a hemisphere (h = base radius r_0).

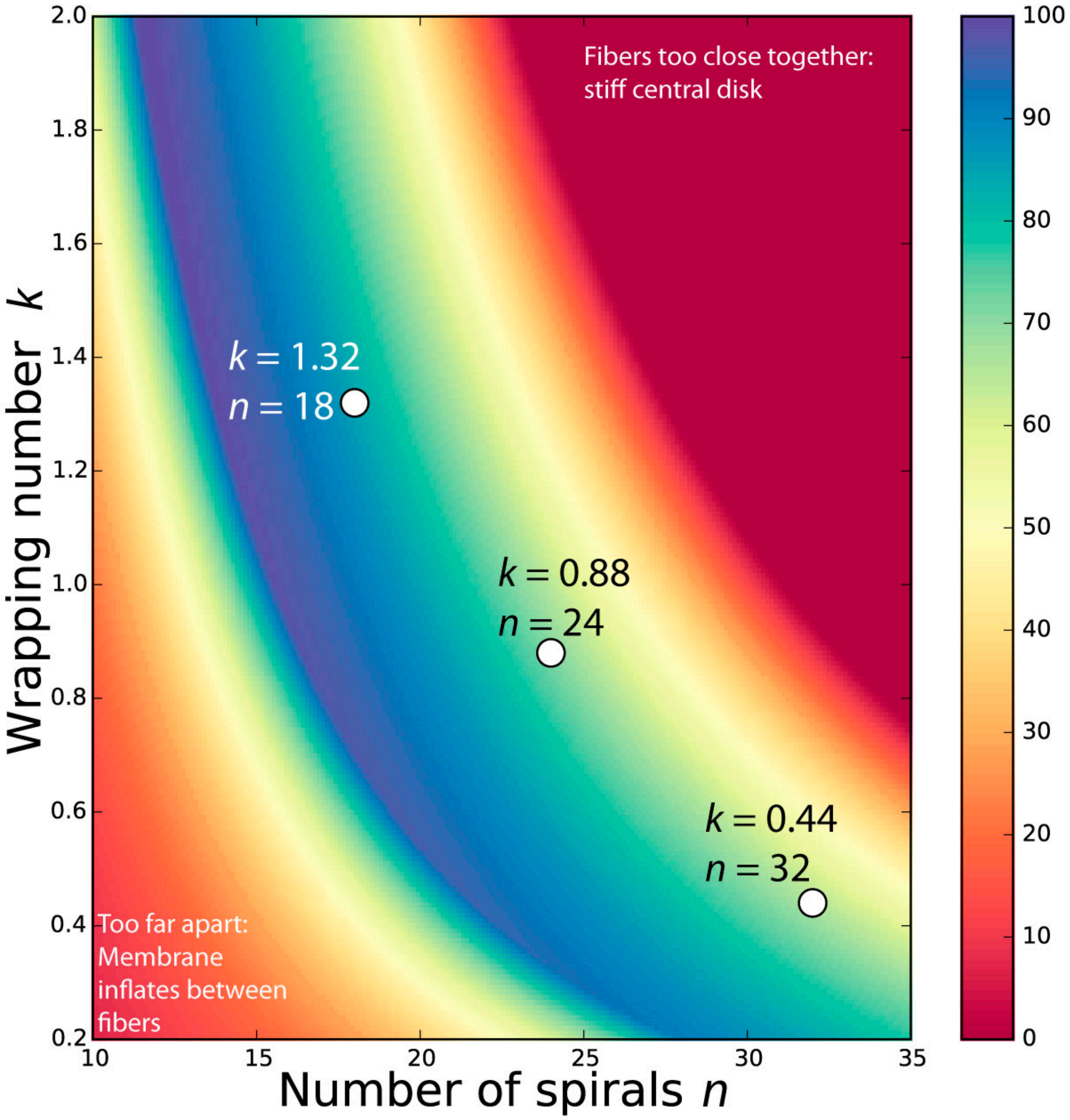

Figure S3. Percentage of membrane that has fiber spacing between one and two times the membrane thickness, for different values of wrap number *k* and number of spiral arms *n*. The three membranes pictured in Figure 2 are marked on the plot, and have 60–70% of their surface area in this "optimal" region where fibers are close enough to constrain the silicone from bulging out between adjacent fibers, but not so close that the fibers stiffen the material. The optimal region always excludes a disk at the center, and sometimes excludes a ring around the edge where the fibers have the greatest separation distance.

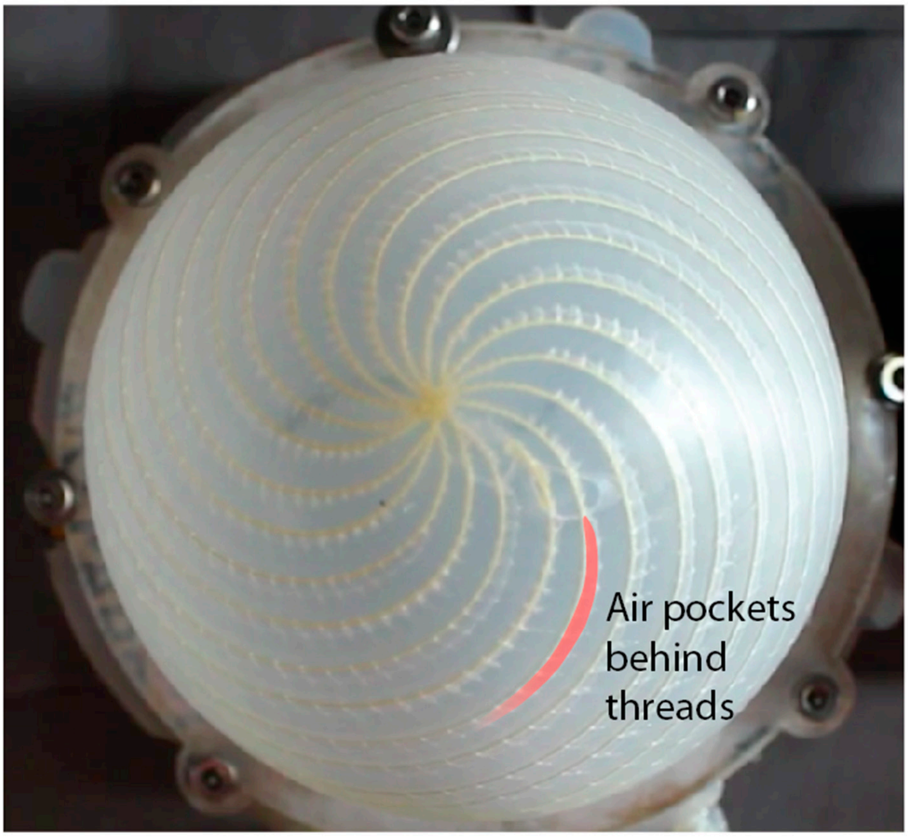

Figure S4. Example of air pockets opening behind threads at high pressures from k = 1.32, pressure = 1.5 psi, actuator at 132° rotation.

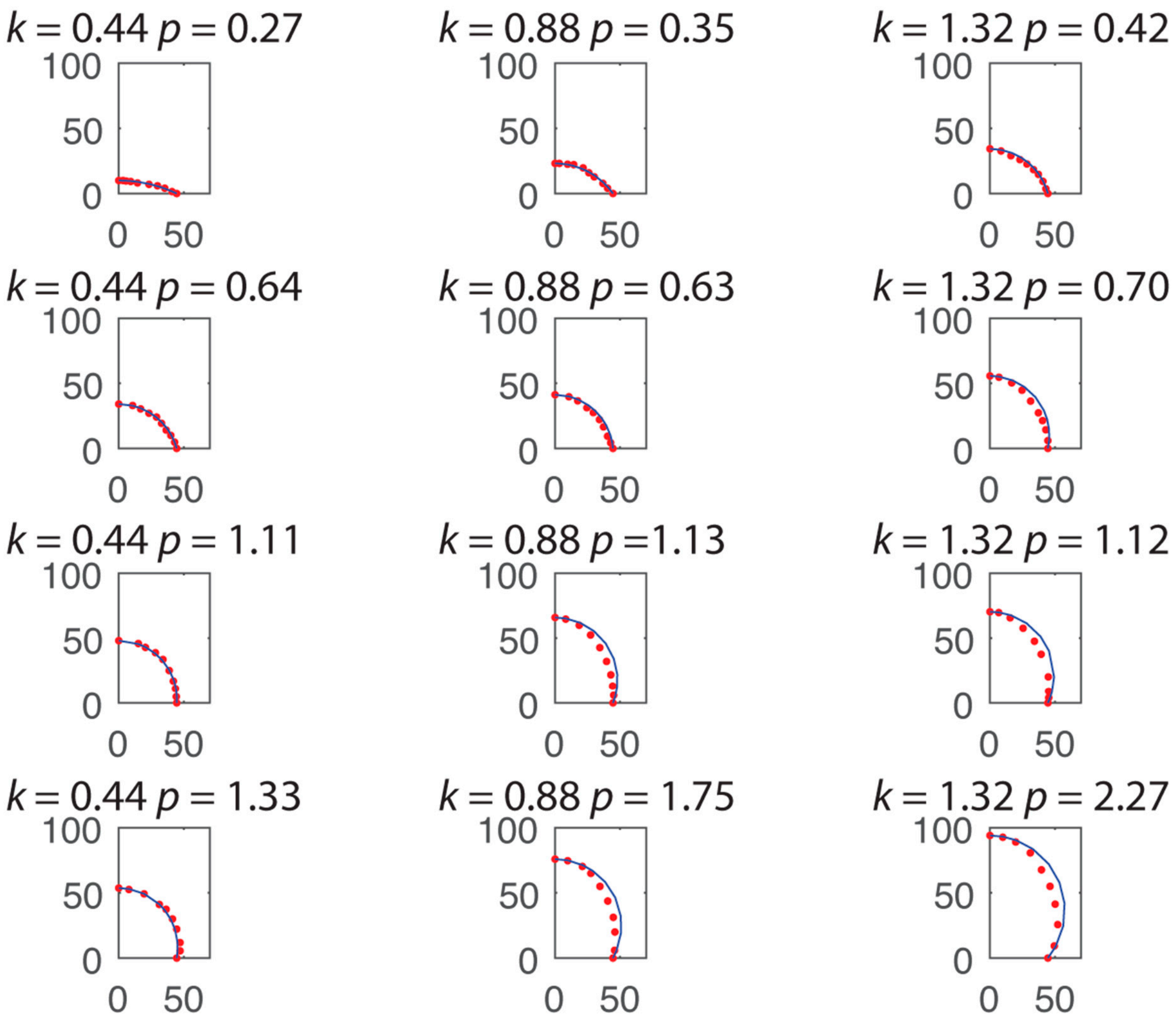

Figure S5. Measured and ideal spherical cap cross-sections of inflated membranes for three *k*-values at increasing pressures (*p*) in psi. The top row and bottom row sample the low-end and high-end pressures for each actuator's full range of motion, respectively, while the middle two rows show how the three different actuators look at similar pressures.

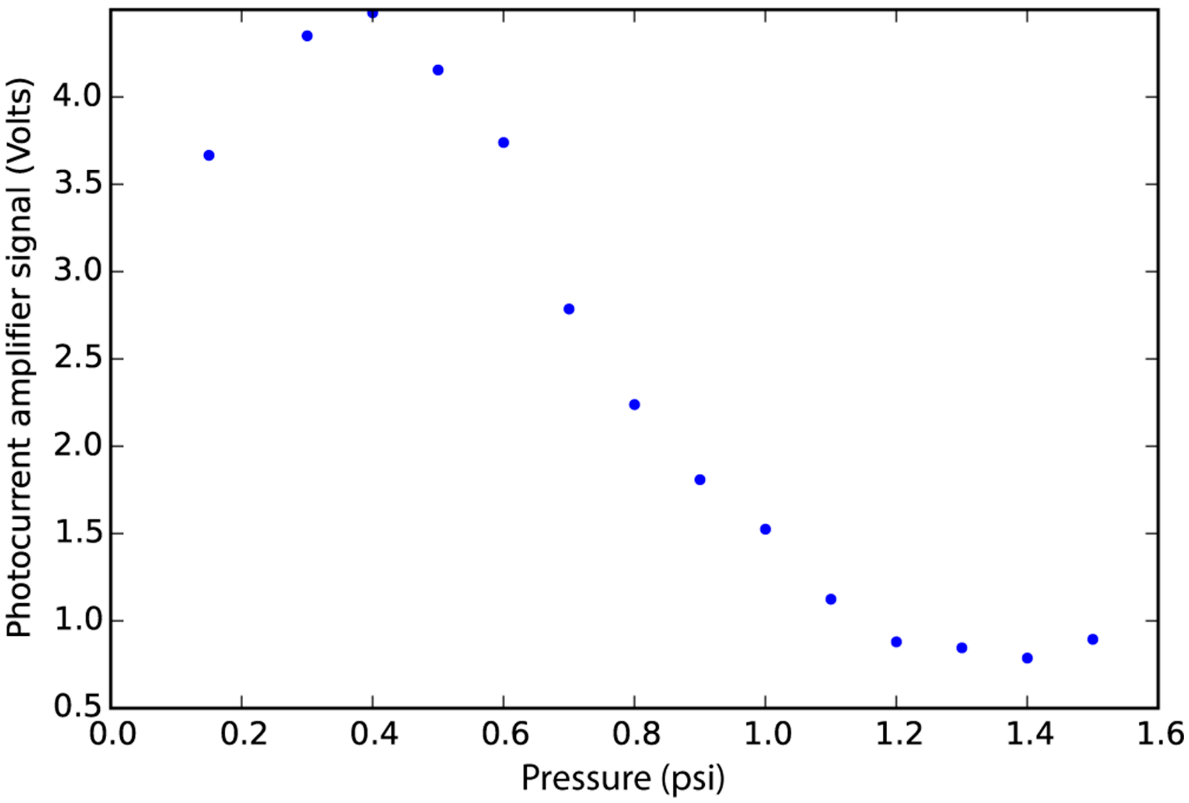

Figure S6. Fiber optic intensity signal vs. pressure for $k = 0.88$, $n = 24$ with straight stretchable optical fiber.

Supplementary Methods

Shape Similarity Metric

Actuator shapes were measured from side-view images and compared to the ideal spherical cap shape using a similarity metric. The similarity metric M for polygons $P1$ (having area $A1$) and polygon $P2$ (having area $A2$) is defined as:

$$M = 1 - \frac{\big((\text{Area of union of } P1 \text{ and } P2) - (\text{Area of intersection of } P1 \text{ and } P2)\big)}{\text{Area of union of } P1 \text{ and } P2} \quad \text{(S1)}$$

For example, if the polygons overlap perfectly, the area of their union and area of their intersection will be the same area $A1 = A2 = A$. The similarity metric becomes $1-(A-A)/A = 1$. If there is no overlap at all, the similarity metric is $1-((A1+A2)-0)/(A1+A2) = 0$. In this work, the measured shape $P1$ is typically smaller than the ideal spherical cap $P2$ and fully contained inside (see Supplementary Figure S5). If a fraction f of the area of $P2$ is filled by $P1$, $M = 1-(A2-fA2)/A2$ or $1-(1-f) = f$. In this case the similarity metric gives the fraction of the ideal spherical cap's 2D cross-sectional area that is filled by the membrane.
